# Tibetan Medicine Qishiwei Zhenzhu Pills Can Reduce Cerebral Ischemia-Reperfusion Injury by Regulating Gut Microbiota and Inhibiting Inflammation

**DOI:** 10.1155/2021/2251679

**Published:** 2021-11-11

**Authors:** Ke Fu, Dewei Zhang, Yinglian Song, Min Xu, Ruixia Wu, Xueqing Xiong, Xianwu Liu, Lei Wu, Ya Guo, You Zhou, Xiaoli Li, Zhang Wang

**Affiliations:** ^1^College of Pharmacy, Chengdu University of Traditional Chinese Medicine, Chengdu 611137, China; ^2^Wanzhou Institute for Drug and Food Control, Chongqing 404000, China; ^3^College of Ethnomedicine, Chengdu University of Traditional Chinese Medicine, Chengdu 611137, China

## Abstract

Cerebral ischemia is a series of harmful reactions, such as acute necrosis of tissue, inflammation, apoptosis, autophagy, and blood-brain barrier injury, due to the insufficient blood supply to the brain. Inflammatory response and gut microbiota imbalance are important concomitant factors of cerebral ischemia and may increase the severity of cerebral ischemia through the gut-brain axis. Qishiwei Zhenzhu pills (QSW) contain more than 70 kinds of medicinal materials, which have the effects of anti-cerebral infarction, anti-convulsion, anti-dementia, and so on. It is a treasure of Tibetan medicine commonly used in the treatment of cerebral ischemia in Tibetan areas. In this study, we gave rats QSW (66.68 mg/kg) once by gavage in advance and then immediately established the rat middle cerebral artery occlusion (MCAO) model. After 24 hours of treatment, the neuroprotection, intestinal pathology, and gut microbiota were examined. The results showed that QSW could significantly reduce the neurobehavioral abnormalities and cerebral infarction rate in MCAO rats. Furthermore, qPCR, western blot, and immunohistochemistry results showed that QSW could effectively inhibit IL-6, IL-1*β*, and other inflammatory factors so as to effectively reduce the inflammatory response of MCAO rats. Furthermore, QSW could improve intestinal integrity and reduce intestinal injury. 16S rRNA sequencing showed that QSW could significantly improve the gut microbiota disorder of MCAO rats. Specifically, at the phylum level, it can regulate the abundance of *Firmicutes* and *Proteobacteria* in the gut microbiota of rats with MCAO. At the genus level, it can adjust the abundance of *Escherichia* and *Shigella*. At the species level, it can adjust the abundance of *Lactobacillus johnsonii* and *Lactobacillus reuteri*. All in all, this study is the first to show that QSW can reduce the severity of cerebral ischemia-reperfusion injury by regulating gut microbiota and inhibiting the inflammatory response.

## 1. Introduction

Cerebral ischemia (CI) or ischemic stroke is due to insufficient blood supply to the brain, which causes hypoxia or ischemia in some areas [1]. Cerebral ischemia-reperfusion injury (I/R) refers to the damage of brain cells caused by CI. The brain is extremely sensitive to hypoxia or ischemia because it receives approximately 750–1,000 mL of blood flow every minute, and its oxygen consumption accounts for approximately 20% of the total for the entire human body. In China, the number of CI cases accounts for approximately 17% to 55% of stroke cases [2], a common clinical cardiovascular and cerebrovascular disease with a fatality rate of more than 40% within 1 month; however, effective treatments are still lacking [3]. Studies have shown that peripheral white blood cells, including T cells, B cells, and neutrophils, can also aggravate the symptoms of CI in the early stage of stroke and release proinflammatory mediators [4]. These immune cells and cytokines can interact not only with each other but also with brain cells. In addition, some clinical studies have found that metabolic syndrome patients with ischemic stroke have higher levels of inflammatory markers and arterial stiffness index than healthy people [5]. All of these studies suggest that inflammation plays a key role in tissue damage after CI.

Gut microbiota are trillions of microorganisms that exist in the human gastrointestinal tract and are inextricably linked to the human body [6]. The microorganisms in the gut microbiota contain genes that are more than 100 times that of the human nuclear genome [7]. These species live in the intestine and perform physiological functions that are essential to humans. Symbiotic physiology occurs between the host and the gut microbiome [8, 9]. The gut microbiota regulates the immune and metabolic balance in the human intestine [6] and can be roughly divided into three categories according to their functions: beneficial, harmful, and conditional pathogenic bacteria. Beneficial bacteria (30%), which include *Lactobacillus*, *Eubacteria*, *Bacillus vulvae*, *Peptococcus*, *Clostridium lecani*, *Cocci*, *Clostridium butyricum*, and *Rossella*, mainly promote gastrointestinal peristalsis and absorption and vitamin synthesis. These organisms can also promote the excretion of harmful substances to the body and protect the body from pathogens. Harmful bacteria (10%), including *Escherichia coli*, *Staphylococcus*, *Streptococcus*, *Clostridium*, *Tetanus*, and *Bacteroides*, can produce harmful substances, increase the intestinal reabsorption of harmful substances, and lead to intestinal abnormal peristalsis, which makes the body vulnerable to pathogen invasion. Conditional pathogenic bacteria (60%), including *E. coli*, *Bacteroides*, *Desulfovibrio*, *Candida albicans*, *Pseudomonas aeruginosa*, and *Proteus*, live in harmony with the human body under normal conditions and will cause harm only under certain conditions [10]. The microbiota-gut-brain axis is a two-way communication network [11]. Several signal molecules (such as catecholamines, serotonin, dynorphin, and cytokines) used by the host for nerve and neuroendocrine signal transduction may also be secreted into the intestinal lumen by neurons, immune cells, and enterochromaffin cells for the gut-brain bidirectional communication [12].

Qishiwei Zhenzhu pills (QSW) are a commonly used traditional Tibetan medicine for treating “bai mai” disease in Tibetan areas, are included in the Pharmacopoeia of the People's Republic of China (2015 edition, Vol. I), and have anti-cerebral infarction [13], anti-convulsant [14], anti-dementia [15], and other effects. This medicine was first published in the Tibetan medicine classic Si Bu Yi Dian and is mainly used for the treatment of “bai mai” disease, stroke, paralysis, hemiplegia, cerebral hemorrhage, and other diseases [16, 17]. This recipe contains more than 70 kinds of medicinal materials, such as myrobalan, pearl, agate, opal, bezoar, coral, musk, gold, silver, and Zuotai [18]. Given its broad range of ingredients, the research on the material basis of its medicinal effects is relatively complicated. The Tibetan medicine Zuotai is the main component of many precious Tibetan medicine preparations (including QSW) and is mainly composed of mercury and other metal elements.

Our recent analysis of 18 elements in QSW revealed the majority of minerals are not absorbed and excreted in the feces, implying the importance of gut-brain axis in QSW-mediated neuroprotection [19]. Therefore, QSW, one of the most effective drugs for the treatment of CI/stroke in Tibetan medicine, could affect microbiota as a mechanism of action. An in-depth study of its action toward CI, inflammation, and microbiota will help provide a clear and deep understanding of QSW in the prevention and treatment of CI. A rat middle cerebral artery occlusion (MCAO) model was used to verify neuroprotective effects of QSW, and a panel of inflammatory mediators were examined via qPCR, western blot, and immunohistochemistry. 16S rRNA sequencing was used to analyze the effects of QSW on the gut microbiota of MCAO rats. The results clearly demonstrated the protective effects of QSW are related to its anti-inflammatory properties and importantly to its effects on gut microbiota regulation.

## 2. Materials and Methods

### 2.1. Drugs and Reagents

QSW (18055A) was purchased from Tibet Ganlu Tibetan Medicine Co. Ltd. Nimodiping (Nmdp, BJ45200) was purchased from Bayer medical and health care Co. Ltd. MN Nucleo Spin 96 Soil Kit, KOD FX Neo, and KOD FX Neo Buf (2X) were bought from TOYOBO. Animal Total RNA Isolation Kit was acquired from FOREGENE Company. Anti-IL-6 and IL-1*β* rabbit antibodies were purchased from Britain Abcam (Shanghai) Trading Co. Ltd. Anti-*β*-actin rabbit antibody was bought from Wuhan Aibotech Biotechnology Co. Ltd. Other chemicals, solvents, and reagents are of analytical grade.

### 2.2. Experimental Animal Model and Design

**Figure 1:**
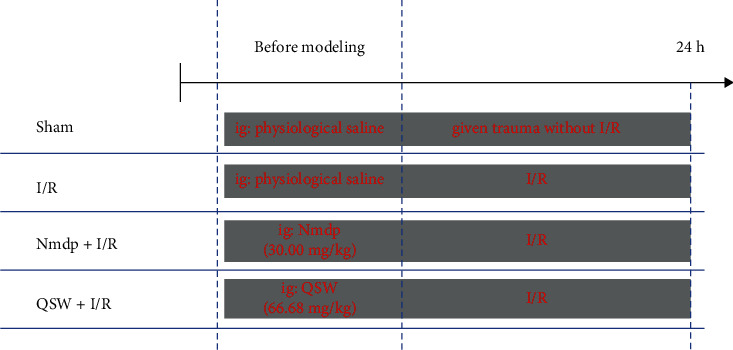
Experimental design.

All Sprague–Dawley (SD) rats of SPF grade were obtained from Chengdu Dashuo Experimental Animal Co. Ltd. (license number: SCXK (Sichuan) 2015-030, experimental animal quality certificate number: 51203500008742). The SD rats were bred in an environment with a 12:12 h light/dark cycle, a room temperature of 25 ± 2°C, and 50 ± 5% humidity. SD rats weighing 250 to 280 g were randomly divided into four groups as follows (*n* = 6/group; Figure 1): Sham, I/R, Nmdp (30 mg/kg, po) + I/R group, and QSW (66.68 mg/kg, po) + I/R group. The selected dose refers to the dose used in the previous experiment of the research group [20–22]. The experiment was conducted in the National Medicine Resource Evaluation Laboratory of Chengdu University of Traditional Chinese Medicine (the third-level scientific research laboratory of the State Administration of Traditional Chinese Medicine, NO. TCM-2009-320). The animal study was reviewed and approved by the Animal Care and Use Committee of Chengdu University of Traditional Chinese Medicine.

### 2.3. Middle Cerebral Artery Occlusion (MCAO) Modeling

Anesthetized rats were cut along the midline of the rat neck, and the myofascial membranes were isolated obtuse along the sternocleidomastoid muscle. The right common carotid artery (CCA), external carotid artery (ECA), and internal carotid artery (ICA) were separated, and ECA was litigated. The pterygopalatine artery inward along the ICA were separated and ligated at the bifurcation. An incision between the distal and proximal ends of the ECA was cut; the tie wire was inserted into the ICA about 20 mm; and the wound was sutured and sterilized. The Sham group only prepared sutures and did not make a model [23]. Following 2 h of CI in rats, the suture was slowly removed to achieve reperfusion. The neck wound was subsequently sutured, and iodophor disinfection was performed. Thereafter, rats were returned to the cage.

### 2.4. Neurological Deficit Score

Reflecting the neurological deficits, the neurobehavioral scores of the experimental rats were measured at 24 hours after CI; the five-level method was used [24, 25]: the first level (−) is no symptoms of nerve injury, the second level (+) is the inability to fully extend the left front paw, the third level (++) is turning to the left, the fourth level (+++) is dumping to the left, and the fifth level (++++) is unable to walk spontaneously and loss of consciousness.

### 2.5. Cerebral Infarction Ratio

Twenty-four hours after CI, rats were anesthetized, and blood was taken from the abdominal aorta and sacrificed. The process is as follows: Take out the whole brain, absorb the moisture on the surface and weigh it, freeze it in the refrigerator at −20°C for 20–30 min, cut it into five evenly with a blade, dye it with the preprepared 1% TTC dye solution at 30°C for 30 min, then separate the infracted part (the pale part), use filter paper to absorb the surface moisture, and weigh again. Cerebral infarction ratio = weight of cerebral infarction part (g)/brain tissue weight (g) × 100%.

### 2.6. Histological Analysis

After the brain was taken, the ileum, cecum, and colon tissues were taken, washed with running water, rinsed with normal saline, and fixed in 10% neutral formaldehyde for intestinal pathological examination. Three indicators were measured in the ileum, including the height of the villi, the width of the villi, and the depth of the crypts. The measurement indicators of the cecum and colon tissue were the thickness of the mucosa.

### 2.7. Gut Microbiota Analysis

While taking the colon, the contents of the colon were collected in a 10 mL dry sterilized centrifuge tube, stored them in an ultralow temperature refrigerator at −80°C, and then sent to Biomarker Technologies (BMK) for 16S rRNA sequencing. Species taxonomy analysis, *α* diversity analysis [26], and species microbial composition structure analysis were performed using BMKCloud (http://www.biocloud.net).

### 2.8. Quantitative Real-Time Polymerase Chain Reaction Analysis

The rats were sacrificed by anesthesia after 24 h of CI, and the brain tissues from the ischemic side were placed in an ultralow temperature refrigerator at −80°C for testing. The RT fluorescent PCR system (total = 20 *μ*L) consists of 2 × Real PCR EasyTM Mix-SYBR (10 *μ*L), forward primer (10 *μ*M, 0.8 *μ*L), reverse primer (10 *μ*M, 0.8 *μ*L), template (DNA) (2 *μ*L), and ddH2O (6.4 *μ*L). Semiquantitative RT-PCR was performed using the following temperature scheme to determine the internal control (*β*-actin): 45 cycles of predenaturation at 95°C for 30 s, denaturation at 95°C for 5 s, annealing at 55°C for 30 s, extension at 72°C for 30 s, and fluorescence collection. The samples were analyzed by RT-PCR amplification curve and melting curve. *F* = 2^−△△CT^ was used to calculate the relative amount of mRNA, △△CT = (average CT value of target gene in drug group − average CT value of internal reference gene in the drug group) − (average CT value of target gene in the blank group − average of reference gene in blank group CT value). The gene primer sequence is listed in Table 1.

### 2.9. Western Blot Analysis

Brain samples were collected from the hippocampus of rats; RIPA lysis solution (brain tissue: lysis solution = 1:10) was added to the brain tissue; the brain samples were cut and placed on ice for 10 min lysis; the lysis solution (12,000 rpm; 4°C) was centrifuged for 10 min; and the supernatant was taken. The further process is as follows: dilute the stock solution, prepare a 1 mg/mL working solution, add 200 µL of BCA working solution, and place it at 37°C for 30 min. Take 50 *μ*g from each experimental group, and add 5× loding buffer at a ratio of 4:1, heat cycle at 95°C for 15 min with a thermal cycler, and store the prepared working solution at −80°C. After sample loading and electrophoresis, membrane transfer, blocking, and antibody culture (primary antibody concentration: Occludin 1:20,000; Claudin-5 1:1,000; MMP-9 1:1,000; *β*-actin 1:5,000), incubate overnight at 4°C. Adding appropriately fluorescently labeled secondary antibodies (dilution concentration 1:5,000), incubate at room temperature for 2-3 h, develop, and fix. Then use the gel imaging system to scan and analyze and express it in terms of the relative expression of the target protein. According to the formula, the relative expression of the target protein = the integral optical density (IOD) of the target protein/IOD of the internal reference for determination.

### 2.10. Immunofluorescence

The fixed tissue was dehydrated, embedded, and sliced by an automatic dehydrator. The dewaxed slices were immersed in 0.01 M citrate buffer (pH 6.0), heated in the microwave oven with high fire until boiling, and then cut off the power. After an interval of 5 min, the chips were repeatedly cooled. The chips were washed 3 times with PBS, 5 minutes each time, 10% serum blocking solution was added, and they were placed in room temperature for 30 minutes. The first antibody was dripped at 4°C overnight; the second antibody was dripped at 37°C for 30 minutes; DAPI was dripped and incubated at room temperature for 10 minutes; and the slices were sealed. The image of the slices was collected by fluorescence scanning microscope camera system, and the fluorescence intensity and area of all the images were measured by the ImageJ analysis system.

### 2.11. Statistical Analysis

The SPSS 21.0 software was applied for data analysis. The data we measured were exhibited by means ± SD. The Student's *t*-test and one-way ANOVA were performed for the comparison between two groups and difference among numerous groups, respectively. Tukey's post hoc tests were applied, for more than two groups. A value of *P* < 0.05 was considered significant statistically.

## 3. Results

### 3.1. QSW Alleviates the Brain Injury Caused by CI in Rats

**Figure 2:**
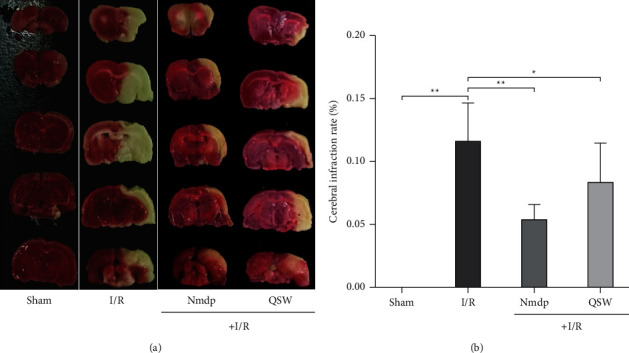
TTC. (a) Representative TTC staining of rat brain slices. Unstained areas indicate tissue damage. (b) QSW alleviates cerebral infarction. The data were presented as means ± SD; *n* = 6 rats per group. Compared with the I/R group, ^*∗*^*P* < 0.05 and ^*∗∗*^*P* < 0.01.

To evaluate the protective effect of QSW on MACO, rats were given QSW in advance, and then a MACO model was established. Compared with those in the Sham group, the rats in the I/R group had neurobehavioral abnormalities at 24 h after MACO (*P* < 0.01). The neurobehavioral abnormalities were significantly improved in the QSW group (66.68 mg/kg) 24 hours after MACO (*P* < 0.05 vs. I/R; Table 2). The results of TTC staining showed that the area of cerebral infarction in rats with MACO could be significantly reduced by QSW (Figure 2(a)). Compared with that of the Sham group, the cerebral infarction rate of rats in the I/R group increased significantly (*P* < 0.01), and the cerebral infarction rate of rats in the QSW group (66.68 mg/kg) was significantly reduced (*P* < 0.05; Figure 2(b)).

### 3.2. QSW Improves Intestinal Integrity in MCAO Rats

**Figure 3:**
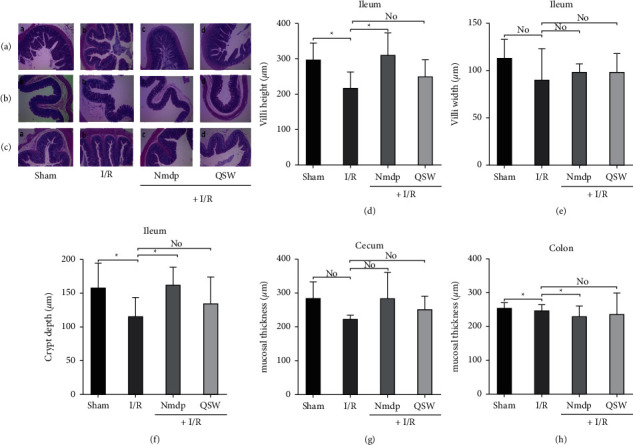
Intestinal damage: (a) H&E staining of the ileum (100× magnification); (b) HE staining of the cecum (100× magnification); (c) HE staining of the the colon (100× magnification); and (d–h) the villi height, villi width, crypt depth, and mucosal layer thickness of the cecum and colon were measured using the BA200 Digital trinocular camera system. The data were presented as means ± SD; *n* = 6 rats per group. Compared with the I/R group, ^*∗*^*P* < 0.05 and ^*∗∗*^*P* < 0.01. “No” means no significant difference.

The H&E staining results showed that the intestinal mucosal structure of MACO rats in the QSW group had a significant change in morphology (Figures 3(a)–3(c)). In three different intestinal regions (ileum, cecum, and colon), compared with the Sham group, the villus height and crypt depth of the ileum in the I/R group were significantly reduced (*P* < 0.05), and the villus width also tended to decrease (*P* > 0.05). The thickness of the mucosa of the cecum did not change significantly (*P* > 0.05), whereas that of the colon was significantly reduced (*P* < 0.05). These results showed that MACO modeling would cause intestinal mucosal damage. Compared with the I/R group, the QSW group showed an increase in the villus height, villus width, crypt depth of ileum, and the mucosal thickness of the cecum and colon (*P* > 0.05). This finding shows that QSW has a certain repairing effect on the intestinal mucosa injury caused by MCAO rats (Figures 3(d)–3(h)).

### 3.3. QSW Changes the Overall Composition of Gut Microbiota

**Figure 4:**
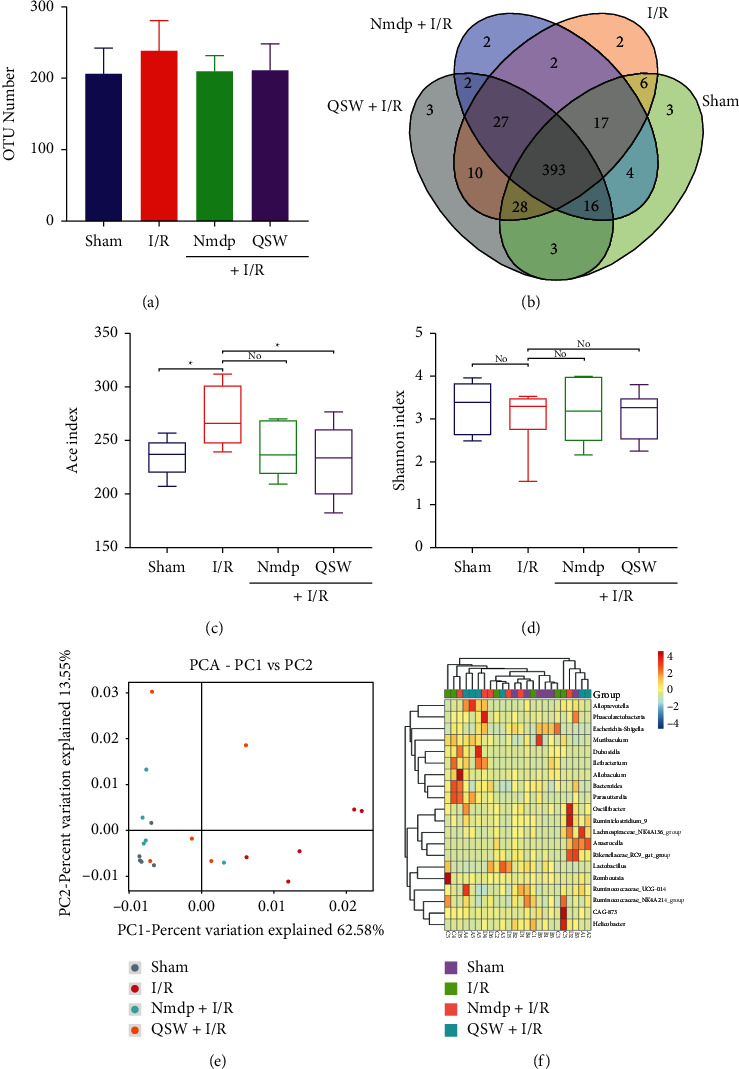
Composition of microbiota: (a) OTU numbers, (b) OTU Venn analysis, (c) Ace index (^*∗*^*P* < 0.05 and ^*∗∗*^*P* < 0.01), (d) Shannon index (^*∗*^*P* < 0.05 and ^*∗∗*^*P* < 0.01), (e) principal component analysis (PCA) for the weighted UniFrac distance of the gut microbiota, and (f) heat map analysis of intestinal flora 16S rRNA detection genus level. The data were presented as means ± SD; *n* = 6 rats per group.

In order to evaluate the effect of QSW on the gut microbiota community, we used 16S rRNA gene amplification and sequence analysis. The result indicated that 146618 sequencing numbers and 518 OTUs were obtained from 24 samples. Compared with the Sham group, the number of OTUs increased (*P* > 0.05) in the I/R group. After QSW pretreatment, the number of OTUs decreased (*P* > 0.05 vs. I/R; Figure 4(a)). Venn diagrams show that there were 393 OTUs in all four groups (Figure 4(b)). According to different alpha diversity indices, as shown in Figures 4(c) and 4(d), compared with the Sham group, the species richness (Ace index) of the I/R group was significantly increased (*P* < 0.05), while the species diversity (Shannon index) was decreased (*P* > 0.05). After pretreatment with QSW, Ace index decreased significantly (*P* < 0.05 vs. I/R), and Shannon index decreased (*P* > 0.05 vs. I/R).

As shown in Figure 4(e), PCA analysis showed that the microbial populations of four groups had different clusters. Compared with the Sham group, the gut microbiota of I/R showed significant structural changes. The total gut microbiota of the QSW group was more similar to that of the Sham group. Additionally, heat map analysis at the genus classification level showed that the composition of gut microbiota in the QSW group was closer to that in the Sham group (Figure 4(f)).

### 3.4. QSW Regulates Gut Microbiota Composition in MCAO Rats

**Figure 5:**
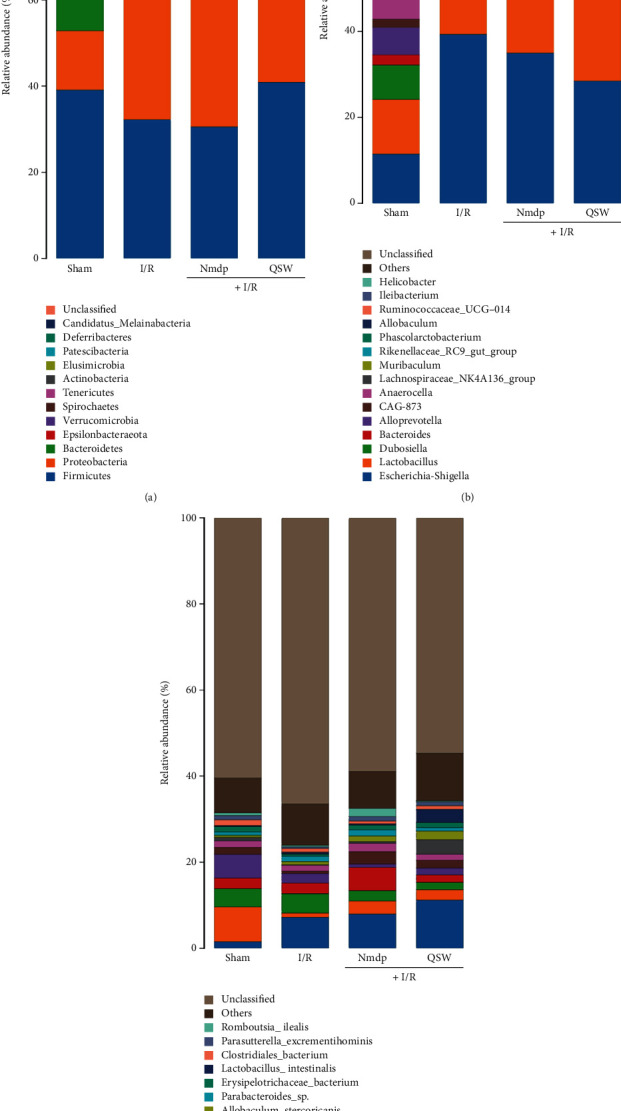
The relative abundance of gut microbiota: (a) phylum level, (b) genus level, and (c) species level.

**Figure 6:**
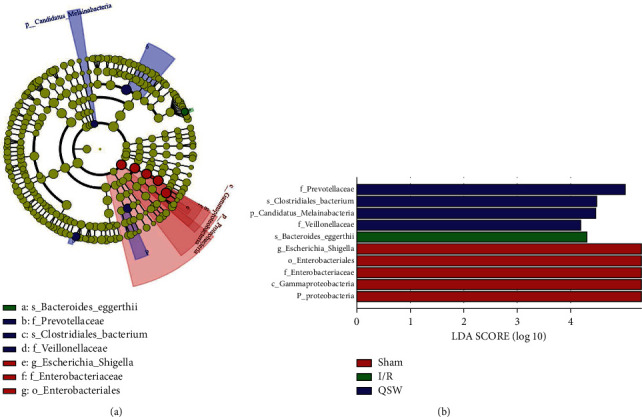
LEfSe analysis comparing differences in the gut microbiota among groups. Species with no significant difference are uniformly yellow, and other colored dots indicate different species in the group.

Next, at the level of phylum classification, *Firmicutes*, *Proteobacteria*, and *Bacteroidetes* are the dominant phyla in the gut microbiota of the four groups of rats. The remaining phyla are *Verrucomicrobia*, *Epsilonbacteraeota*, *Spirochaetes*, *Tenericutes*, *Actinobacteria*, *Patescibacteria*, and *Elusimicrobia*. Compared with the Sham group, after successful MCAO, the abundance of *Firmicutes* and *Bacteroidetes* in the gut microbiota of rats decreased, while the abundance of *Proteobacteria* increased. When MCAO model rats were treated with QSW, the abundance of *Firmicutes* in the gut microbiota of rats increased, while the abundance of *Proteobacteria* is reduced (Figure 5(a)). At the taxonomic level of genus, *Escherichia Shigella* and *Lactobacillus* are the dominant bacterial genera in the gut microbiota of the four groups of rats. Meanwhile, the remaining genera are *Bacteroides*, *Alloprevotella*, *Phascolarctobacterium*, *Rikenellaceae-RC9-gut-group*, *Anaerocella*, *Dubosiella*, *CAG-873*, and *Allobaculum*. Compared with the Sham group, after successful MCAO, the abundance of *Escherichia Shigella* in the gut microbiota of rats increased significantly. When MCAO model rats were given QSW, the abundance of *Escherichia Shigella* in the gut microbiota of rats is reduced (Figure 5(b)). What's more, at the level of species classification, the four groups of rat gut microbiota with relative abundance greater than 0.1% are exemplified, so *Lactobacillus johnsonii*, *Firmicutes bacterium*, *Lactobacillus reuteri*, and *Bacteroidales bacterium* are the dominant strain in the gut microbiota of the four groups of rats. Compared with the Sham group, after successful MCAO modeling, the abundance of *F. bacterium* in the gut microbiota of rats was significantly reduced, and the abundance of *L. johnsonii* was significantly increased. When MCAO model rats were given QSW, the abundance of *L. johnsonii* in the gut microbiota of rats increased, and *L. reuteri* abundance decreased (Figure 5(c)). In addition, the LEfSe analysis showed the species with significant differences among the groups (Figure 6).

### 3.5. QSW Attenuates Inflammatory Response in Rats with CI

**Figure 7:**
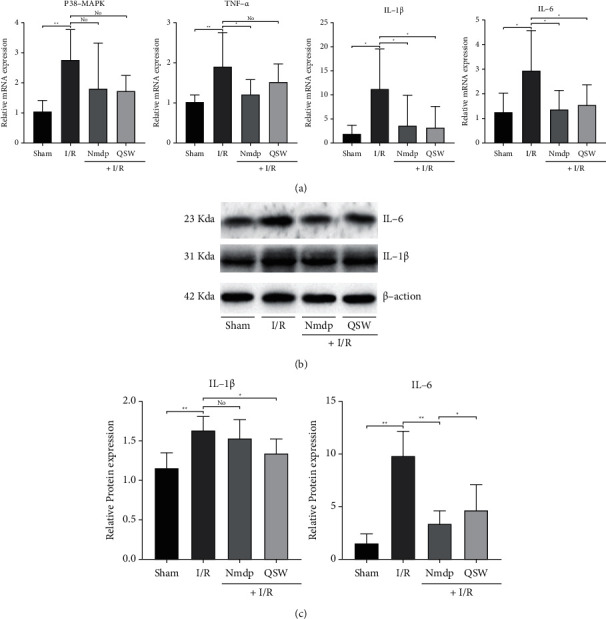
Inflammation: (a) the mRNA levels of p38-MAKP, TNF-*α*, IL-1*β*, and IL-6 in the hippocampus of the ischemic side were measured by RT-PCR; (b) the protein levels of IL-1*β* and IL-6 in the hippocampus of the ischemic side were measured by western blot; and (c) histogram analysis of the levels of IL-1*β* and IL-6. The data were presented as means ± SD; *n* = 6 rats per group. Compared with the I/R group, ^*∗*^*P* < 0.05 and ^*∗∗*^*P* < 0.01. “No” means no significant difference.

**Figure 8:**
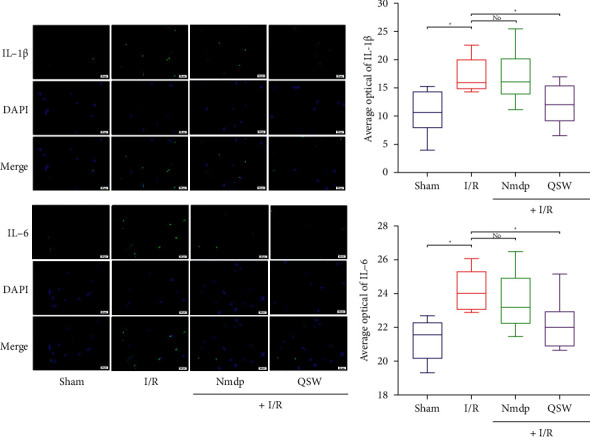
IL-1*β* and IL-6 IHC. Representative microphotographs of immunofluorescence staining (400×) for identification of IL-1*β* and IL-6. The data were presented as means ± SD; *n* = 6 rats per group. Compared with the I/R group, ^*∗*^*P* < 0.05 and ^*∗∗*^*P* < 0.01. “No” means no significant difference.

We measured the expression of inflammatory factors in the tissue of the hippocampus on the ischemic side of the brain. The results showed that QSW could downregulate the expression of p38-MAPK, TNF-*α* (*P* > 0.05) and IL-1*β* and IL-6 (*P* < 0.05) mRNA compared with the I/R group (Figure 7(a)). In addition, we selected IL-1*β* and IL-6 for western blot and immunofluorescence experiment and found that QSW could downregulate the protein expression of IL-1*β* and IL-6 (*P* < 0.05) compared with the I/R group (Figures 7(b), 7(c), and 8). Therefore, we speculate that the QSW may inhibit inflammation and inflammation-related pathways to alleviate the inflammation of MCAO in rats.

## 4. Discussion and Conclusion

The present study confirmed and extended our prior study of the protective effect of QSW on I/R through the blood-brain barrier and metabolomics in the early stage [20]. QSW can protect against lipopolysaccharide plus 1-methyl-4-phenyl-1,2,3,6-tetrahydropyridine-induced neurotoxicity and disturbance of gut microbiota in mice [27]. But so far, there are few reports on the changes of microbiota and the effect of microbiota changes on neuroinflammation during the treatment of I/R with QSW.

In this study, we established the MCAO model and treated it with QSW by gavage. It was found that QSW reduced the neurobehavioral abnormalities and the ratio of cerebral infarction caused by I/R. This may be related to the improvement of microbiota imbalance and intestinal barrier damage by QSW. Our data also showed that QSW reduced IL-6 and IL-1*β* mRNA and protein expression levels, thereby inhibiting inflammation. This study can provide an important theoretical basis for the in-depth study of the anti-CI and central inflammation effects of QSW through the gut-brain axis and its reasonable clinical application.

It is reported that focal and transient MCAO in rodents has been reported to lead to neuropathological results similar to clinical CI. At present, there are methods to prevent cerebral infarction, but there is no way to improve the anatomical, neurochemical, and behavioral defects after CI [28]. Therefore, we established a rat MACO model to evaluate the effects of QSW on cerebral infarction and neuropathology. TTC results showed that QSW could significantly reduce the area of cerebral infarction. At the same time, the TTC experiment can also prove that our rat MACO model is successful. In addition, QSW can significantly improve neurological behavior. In conclusion, QSW can effectively protect the neurobehavioral abnormalities and the increase of cerebral infarction ratio caused by CI.

Gut microbiota is an important part of the intestinal barrier. It is very important for the physiological processes related to the functional maturity of the host intestinal mucosal barrier, nutrient absorption, immune system development, and energy metabolism [29, 30]. Recent evidence shows that the occurrence of CI is closely related to the changes of gut microbiota [31–33]. Therefore, more and more attention has been focused on the regulation of gut microbiota as a therapeutic strategy against CI and related diseases. In this study, we found that QSW can alleviate CI by regulating the composition of gut microbiota. At the phylum level, it can regulate the abundance of *Firmicutes* and *Proteobacteria* in the gut microbiota of rats with MCAO. At the genus level, it can adjust the abundance of *Escherichia and Shigella*. At the species level, it can adjust the abundance of *L. johnsonii* and *L. reuteri*. Cluster classification results also showed that the composition of the gut microbiota of rats in the QSW group was closer to that of the Sham group, suggesting that QSW could improve the environment of the gut microbiota in rats with CI. Besides, evidence suggests that *Helicobacter pylori* is the pathogenic mechanism of CI through its interference with lipid and lipoprotein metabolism and atherosclerosis promotion [34]. Meanwhile, *L. johnsonii* can inhibit *Helicobacter* in vitro and in vivo, and the combined anti-H. *pylori* urease IgY administration suppresses *H. pylori* to a greater extent than the monotherapy against *L. johnsonii* [35]. Therefore, QSW regulating the abundance of *Lactobacillus yoelii and L*. *reuteri* is possibly one of the mechanisms of treatment for CI and is probably related to *L. yoelii* control.

Inflammation is closely related to central nervous system diseases. Evidence indicates that the physiological basis of the inflammatory response in the central nervous system is the activation of microglia and astrocytes and the exudation of leukocytes [36]. Under the pathological conditions of CI and stroke, microglia and astrocytes secrete a large number of inflammatory factors, such as IL-1, TNF-*α*, NF-*κ*B p65, ICAM-1, and other inflammatory factors that play a huge role in the inflammatory cascade [37, 38]. What's more, inhibition of the activation of p38MAPK signal pathway not only can reduce the neuronal damage by blocking the proapoptotic pathway but also can block the inflammatory signal-mediated effect [39]. In addition, a recent report showed that the imbalance of gut microbiota caused by antibiotic treatment affects the prognosis of poststroke neuroinflammation in experimental stroke models [32]. In another report, acute brain injury can cause gut microbiota imbalance, and gut microbiota is a key regulator of neuroinflammatory response after brain injury [40]. These findings highlight the key role of the microbiota as a potential therapeutic target to protect the normal function of the brain after injury. In this study, we found QSW can adjust the disordered gut microbiota to a balanced state. Besides, the expression of IL-6 and IL-1*β* mRNA in the ischemic lateral brain tissue of the QSW group decreased significantly, while the expression of p38-MAPK and TNF-*α* mRNA decreased. In addition, we further studied IL-6 and IL-1*β* western blot, and immunofluorescence experiments confirmed our PCR results.

In conclusion, the present study demonstrated that QSW can reduce the severity of I/R in rats by regulating gut microbiota and inhibiting the inflammatory response.

## Figures and Tables

**Table 1:** Primers for real-time PCR.

Gene	Forward primer (5′-3′)	Reverse primer (5′-3′)
IL-6	ACAGAGGATACCACCCACAACAGACC	CGGAACTCCAGAAGACCAGAGCAGAT
IL-1*β*	ATCCTCTCCAGTCAGGCTTCCTTGTG	AGCTCTTGTCGAGATGCTGCTGTGA
p38-MAPK	GACGAATGGAAGAGCCTGACCTACGA	TGGACAAACGGACAGACAGACAGACA
TNF-*α*	CCAGCCAGGAGGGAGAACAGCAACT	CCGCCACGAGCAGGAATGAGAAGAG

**Table 2:** QSW on behavioral scores.

Group	Dose (mg·kg^−1^)	Neurobehavioral at 24 h					
		−	+	++	+++	++++	*P* value
Sham	—	6	0	0	0	0	0.0017^*∗∗*^
I/R	—	0	0	2	4	0	—
Nmdp + IR	30.00	0	0	5	1	0	0.0419^*∗*^
QSW + IR	66.68	0	0	6	0	0	0.0017^*∗∗*^

Compared with the I/R group, ^*∗*^*P* < 0.05 and ^*∗∗*^*P* < 0.01.

## Data Availability

The data supporting the conclusions are included in the manuscript. The data sets used and/or analyzed during the current study are available from the corresponding author on reasonable request.
